# Susceptibility of Hamsters to *Clostridium difficile* Isolates of Differing Toxinotype

**DOI:** 10.1371/journal.pone.0064121

**Published:** 2013-05-21

**Authors:** Anthony M. Buckley, Janice Spencer, Lindsay M. Maclellan, Denise Candlish, June J. Irvine, Gillian R. Douce

**Affiliations:** Institute of Infection, Immunity and Inflammation, Glasgow Biomedical Research Centre, University of Glasgow, Glasgow, United Kingdom; Institute Pasteur, France

## Abstract

*Clostridium difficile* is the most commonly associated cause of antibiotic associated disease (AAD), which caused ∼21,000 cases of AAD in 2011 in the U.K. alone. The golden Syrian hamster model of CDI is an acute model displaying many of the clinical features of *C. difficile* disease. Using this model we characterised three clinical strains of *C. difficile*, all differing in toxinotype; CD1342 (PaLoc negative), M68 (toxinotype VIII) & BI-7 (toxinotype III). The naturally occurring non-toxic strain colonised all hamsters within 1-day post challenge (d.p.c.) with high-levels of spores being shed in the faeces of animals that appeared well throughout the entire experiment. However, some changes including increased neutrophil influx and unclotted red blood cells were observed at early time points despite the fact that the known *C. difficile* toxins (TcdA, TcdB and CDT) are absent from the genome. In contrast, hamsters challenged with strain M68 resulted in a 45% mortality rate, with those that survived challenge remaining highly colonised. It is currently unclear why some hamsters survive infection, as bacterial & toxin levels and histology scores were similar to those culled at a similar time-point. Hamsters challenged with strain BI-7 resulted in a rapid fatal infection in 100% of the hamsters approximately 26 hr post challenge. Severe caecal pathology, including transmural neutrophil infiltrates and extensive submucosal damage correlated with high levels of toxin measured in gut filtrates *ex vivo*. These data describes the infection kinetics and disease outcomes of 3 clinical *C. difficile* isolates differing in toxin carriage and provides additional insights to the role of each toxin in disease progression.

## Introduction

Since its first association with antibiotic-associated disease (AAD) in 1977, *Clostridium difficile* has been recognised as the most commonly identified cause of nosocomial diarrhoea world-wide [1]. *C. difficile* infection (CDI) typically occurs following antibiotic therapy, in which disruption of the resident gut flora leaves the intestine susceptible to *C. difficile* outgrowth. CDI can result in a range of clinical sequelae; asymptomatic carriage to severe diarrhoea, pseudomembranous colitis and death. In the U.K. cases of CDI peaked in 2007 at 57,255 cases but, more recently CDI rates have declined to 21,682 in 2011, presumably due to the modification of antibiotic use and the implementation of improved containment barrier protocols [2, www.hpa.org.uk & www.hps.scot.nhs.uk]]. In the US, *C. difficile* is associated with over 14,000 deaths annually [www.cdc.gov/HAI/organisms/cdiff updated 2010]. The total identifiable cost of CDI was estimated to be £4000 per case in England in 1996 [3]. On this basis such infections conservatively cost the U.K. £87 million in 2011.

Disease has largely been associated with the production of two large toxins, toxin A (TcdA) and toxin B (TcdB), which are encoded by *tcdA* and *tcdB* (respectively) along with three other genes on the pathogenicity locus (PaLoc) [4]. Variations within this locus are recognised by a typing scheme, which recognises at present 31 different toxinotypes [5], [6]. These include the toxinotype III group to which Ribotype 027 isolates [7] have been associated with global CDI increase. These strains typified by the NAP01/Ribotype 027 isolates (A+B+CDT+) were responsible for 41.3% of all CDI cases between 2007–2008 in the U.K. [8]. Ribotype 027 isolates have been associated with increased disease severity (such as toxic megacolon) and relapse rates [9]. Another toxinotype that has also been associated with a global increase of CDI outbreaks, especially in Asia, are the A−B+ toxinotypes (types VIII, X, XVI, XVII, XXX & XXXI). In 2008 the proportion of A−B+ isolates recovered from Korean CDI cases was 25.7% compared to 4.2% of isolates recovered in 1995 [10], [11]. After initially being thought as non-pathogenic it is now known that A−B+ toxinotypes can cause a wide spectrum of disease including pseudomembranous colitis and mortality [12], [13]. Toxin A−B+ isolates have typically been typed as Ribotype 017, however, in recent years some A−B+ strains isolated in China & Australia appear as distinctly separate ribotypes [14], [6]. Along with toxigenic strains, naturally occurring non-toxigenic (A−B−) *C. difficile* are associated with asymptomatic carriage in both adults and infants. In these strains the PaLoc region encoding the toxins is replaced by a short sequence of 115 bp. Non-toxigenic carriage rates depend on age with several reports highlighting infants (≤2 years-old) and the elderly as sources of community *C. difficile*, with carriage rates as high as 26.5% [15] and 2.7% [16], respectively.

Sporulation, which is the transformation of vegetative cells to metabolically dormant endospores, is an important process for *C. difficile* transmission. As *C. difficile* spores are shed in the faeces of CDI patients, any surface or device contaminated with faeces can act as a reservoir for infection [17]. *C. difficile* spores are highly resistant to several hospital cleaning agents [18], desiccation [19], pH extremes and high temperatures [20]. Deakin *et al.*, [21] elegantly showed that sporulation is essential for a persistent and relapsing disease and that transmission can occur through environmental contamination, direct contact and airborne transmission. *C. difficile* strains that exhibit increased disease severity and relapse are typically associated with increased sporulation rates compared to non-epidemic strains and historical strains [22], [23].

Within this study, we investigated clinical strains that naturally express different combinations of the toxins in the golden Syrian hamster model of disease. This model mirrors many clinical aspects of human CDI, as hamsters pre-treated with clindamycin results in haemorrhagic caecitis, which manifests as ‘wet tail’ and eventually death [1], [24]. In this manuscript we have used this model to characterise the disease outcomes of three clinical *C. difficile* strains; BI-7 – toxinotype III (A+B+CDT+), isolated from an epidemic outbreak in Portland, U.S. 2003, M68 - toxinotype VIII (A−B+CDT−), isolated during a *C. difficile* outbreak in Dublin, Ireland 2004 [12] & CD1342 –PaLoc negative (A−B−CDT−), isolated from an asymptomatic paediatric patient in Oxford, U.K. 2009 [25].

## Materials and Methods

### Bacterial strains and spore preparation

*C. difficile* BI-7 was donated by Dr Trevor Lawley (Wellcome Sanger Institute, Cambridge, U.K.); M68 was obtained from Dr Richard Stabler (London School of Hygiene and Tropical Medicine, London, U.K.) and CD1342 was obtained from Dr Kate Dingle (Oxford University, Oxford, U.K.). BI-7 is Ribotype 027, toxinotype III (A+B+CDT+), M68 is Ribotype 017, toxinotype VIII (A−B+CDT−) [12] and CD1342 is Ribotype 005, toxinotype PaLoc negative (A−B−CDT−) [25]. Strains were grown on CCEY agar supplemented with cefoxitin-cycloserine, egg emulsion (Lab M, Lancaster, U.K.) and erythromycin (100 mg/L; M68 & CD1342) or clindamycin (20 mg/L; BI-7) at 37°C under anaerobic conditions. Animal inoculation spores were made according to Buckley *et al.* [24] and were enumerated, to calculate the inoculating dose, by 10-fold serial dilutions plated onto supplemented CCEY agar plates as above.

### Minimum inhibitory concentration (MIC)

The MIC of *C. difficile* BI-7, M68 and CD1342 to erythromycin and clindamycin was determined by the broth doubling dilution method as described by Andrews [26]. Briefly, rows of pre-conditioned BHI broth (90 µl) were supplemented with a concentration range of 1024–0.5 µg/ml of either antibiotic. Wells were inoculated with ∼5000 spores/100 µl (as determined from spore preparations above) and incubated at 37°C for 48 h anaerobically. A no antibiotic positive control row was setup alongside an uninoculated sterility control row. After incubation, plates were visually inspected and compared to the controls; the MIC end point was determined as the lowest concentration of antibiotic at which there is no visible growth. Results are given as the median MIC from at least three assays.

### Ethics statement

All procedures were strictly conducted according to the requirements of the Animals (Scientific Procedures) Act 1986 approved by the Home Office, U.K. (project licence 60/4218).

### Animal experiments

All hamster procedures, including telemetry chip insertion, clindamycin dosing and *C. difficile* challenge, were done following to Buckley *et al.* [24]. For survival assays, animals were monitored for any signs of morbidity, including ‘wet tail’. Animals were culled if core body temperature fell below 35°C (previously shown to be a relevant and effective endpoint). *C. difficile* faecal shedding was monitored in those animals that survived challenge. When an animal succumbed to infection, to establish level of colonisation, the caecum and colon were removed aseptically at *post-mortem*. To enumerate the total bacterial load (spores and vegetative cells), each section was opened longitudinally, and the contents were removed by gentle washing in 10 ml PBS (luminal associated bacteria). The tissues washed in 10 ml PBS and homogenized in 5 ml of PBS for 1 min (tissue associated bacteria), and viable counts were determined for the homogenates. Serial 10-fold dilutions were plated on supplemented CCEY agar plates. To determine the numbers of spores present in the samples, the samples were heated for 15 min at 56°C, and the numbers of spores present were determined by the viable count method as described above. To monitor the bacterial recoveries as the infection progressed, at least 5 animals were each culled at 1-, 3- & 11-days post challenge (d.p.c), where the caecum and colon were excised and processed as above. Results are shown as mean number of recovered bacteria from 2 biological replicates, where a total of at least 5 animals were included per time point. Colonies were confirmed using multilocus variable-number tandem-repeat analysis (MVLA) [27 data not shown].

### Detection of *in vivo* toxin levels

Production of *C. difficile* toxins were detected *in vitro* using filtered caecum content from animals taken during *post mortem* as described by Buckley *et al.*, [24]. Briefly, monolayers of Vero cells (kidney epithelial cells) were washed with preheated sterile PBS before addition of serial diluted filtered gut content in supplemented EMEM and incubated for 18 h at 37°C (5% CO_2_). Cells were washed with PBS, fixed in 1% formalin for 10 min then washed again. Fixed adherent cells were stained with Geimsa for 30 min then washed before addition of 0.1% SDS and left for 1 h. Optical density was taken using an EL_x_808 Ultra microplate reader (Bio-Tek instruments) at 600 nm and compared to non-infected hamster caecal and colon gut contents as a negative control. If the toxin dilution was able to cause cell toxicity (cell rounding) this leads to the loss of cell adherence resulting in a reduced staining and hence optical density of the wells. Results are expressed as LOG reciprocal titre.

### Histology

Caecum samples were prepared for simple histology as described by Goulding *et al*, [28]. Caecal pathology was scored in a blinded fashion, grading neutrophil margination (0, no neutrophil accumulation; 1, local acute neutrophil accumulation; 2, extensive submucosal neutrophil accumulation; 3, transmural neutrophilic infiltrate), haemorrhagic congestion (0, normal tissue; 1, engorged mucosal capillaries; 2, submucosal congestion with unclotted blood; 3, transmural congestion with unclotted blood), hyperplasia (0, no epithelial hyperplasia; 1, twofold increase in thickness; 2, threefold increase in thickness; 3, fourfold or greater increase in thickness), and percent of epithelial barrier involvement (0, no damage; 1, less than 10% of mucosal barrier involved; 2, less than 50% of mucosal barrier involved; 3, more than 50% mucosal barrier involved). Results are expressed as mean pathology score per strain for each criterion.

### Statistical analysis

All statistical analyses were performed using the GraphPad Instat 3.10 (GraphPad Instat Software). A Mann-Whitney analysis of variance analysis (ANOVA) was used to determine significant difference in bacterial recoveries between all time points examined. *P* values ≤0.05 were considered significant.

## Results

### Antimicrobial susceptibility

*C. difficile* M68 and CD1342 were highly resistant to both erythromycin and clindamycin, MIC>1024 µg/ml & >256 µg/ml, respectively. Strain BI-7 showed resistance to clindamycin (MIC = 64 µg/ml) but was highly susceptible to erythromycin (MIC = 0.125 µg/ml). Using this data we determined that a one-day clindamycin hamster treatment model could be used without any diminutive effects on the initial inoculum for each strain.

### Telemetry monitoring and survival of infected hamsters

**Figure 1:**
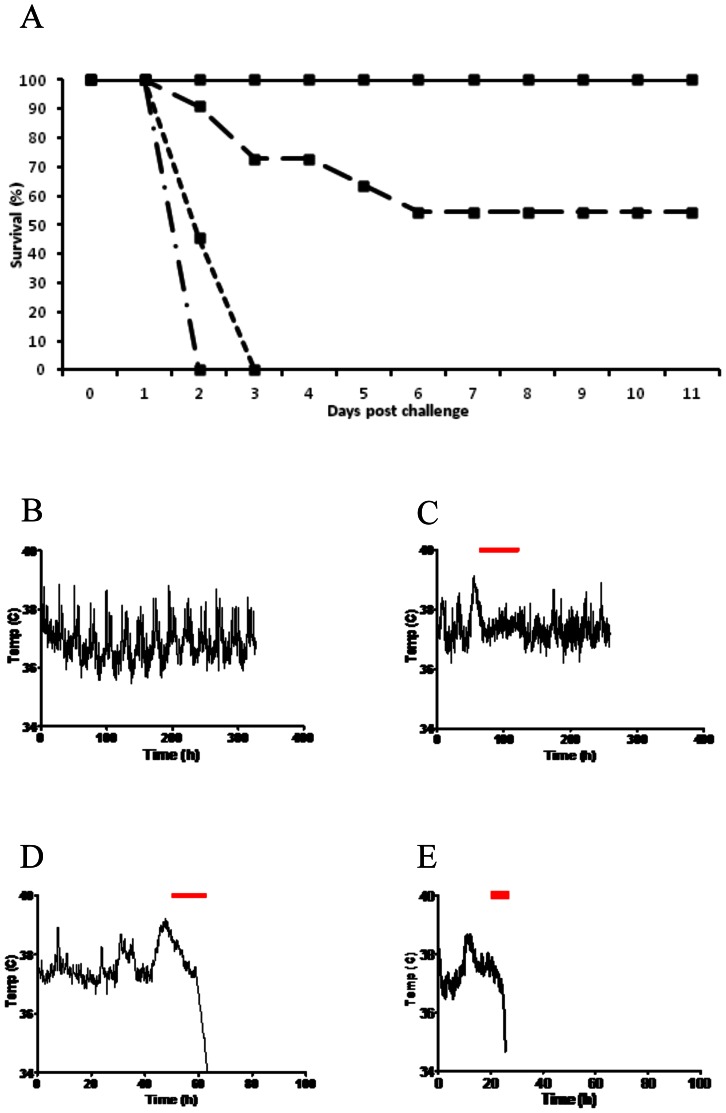
Survival of hamsters challenged with *C. difficile* strains. (A) Survival graph of hamsters challenged with either *C. difficile* CD1342 (full line), M68 (long dashed line), BI-7 (dash/dot line) or R20291 [dotted line (R20291 data from 24]. (B) Typical body temperature kinetics of a hamster challenged with CD1342 following the usual diurnal pattern. (C) Body temperature kinetics of a surviving hamster challenged with M68, (D) a hamster that succumbed to M68 challenge & (E) a hamster challenged with BI-7. Top bar represents when symptoms, typically ‘wet tail’, manifest. A typical febrile response (maximum temperature of 39.3°C) was seen prior to onset of observed symptoms.

Challenge of clindamycin pre-treated hamsters with 10,000 spores of *C. difficile* BI-7, M68 or CD1342 resulted in a 100% colonisation rate. Hamsters challenged with the toxin negative (A−B−) strain CD1342 resulted in a 100% survival rate with no signs of morbidity (Figure 1A & B). In contrast, when challenged with M68 (A−B+) 55% (6/11) of hamsters survived, however all animals showed classical symptoms of *C. difficile* infection, i.e. ‘wet tail’ (Figure 1A & C). Those animals that succumbed to infection with M68 had a mean time to cull of 82.55 h±16.9 h SEM, however a wide range between 43.7 h and 127.0 h was observed (n = 5). An extended period of high body temperature was associated with those hamsters that survived M68 infection, e.g. a peak body temperature of 39.3°C was observed before the onset of symptoms in the surviving hamster profile shown in Figure 1C. In comparison hamsters that succumbed to M68 challenge showed slightly elevated body temperatures before the rapid temperature drop associated with CDI in hamsters (Figure 1D). Animals challenged with BI-7 resulted in a 100% fatal infection with a mean time to cull of 26.3 h±0.75 h SEM. When animals succumbed to infection a rapid temperature drop was observed similar to other ribotype 027 isolates (Figure 1A & E; [24]).

### Faecal shedding of CD1342 & M68

**Figure 2:**
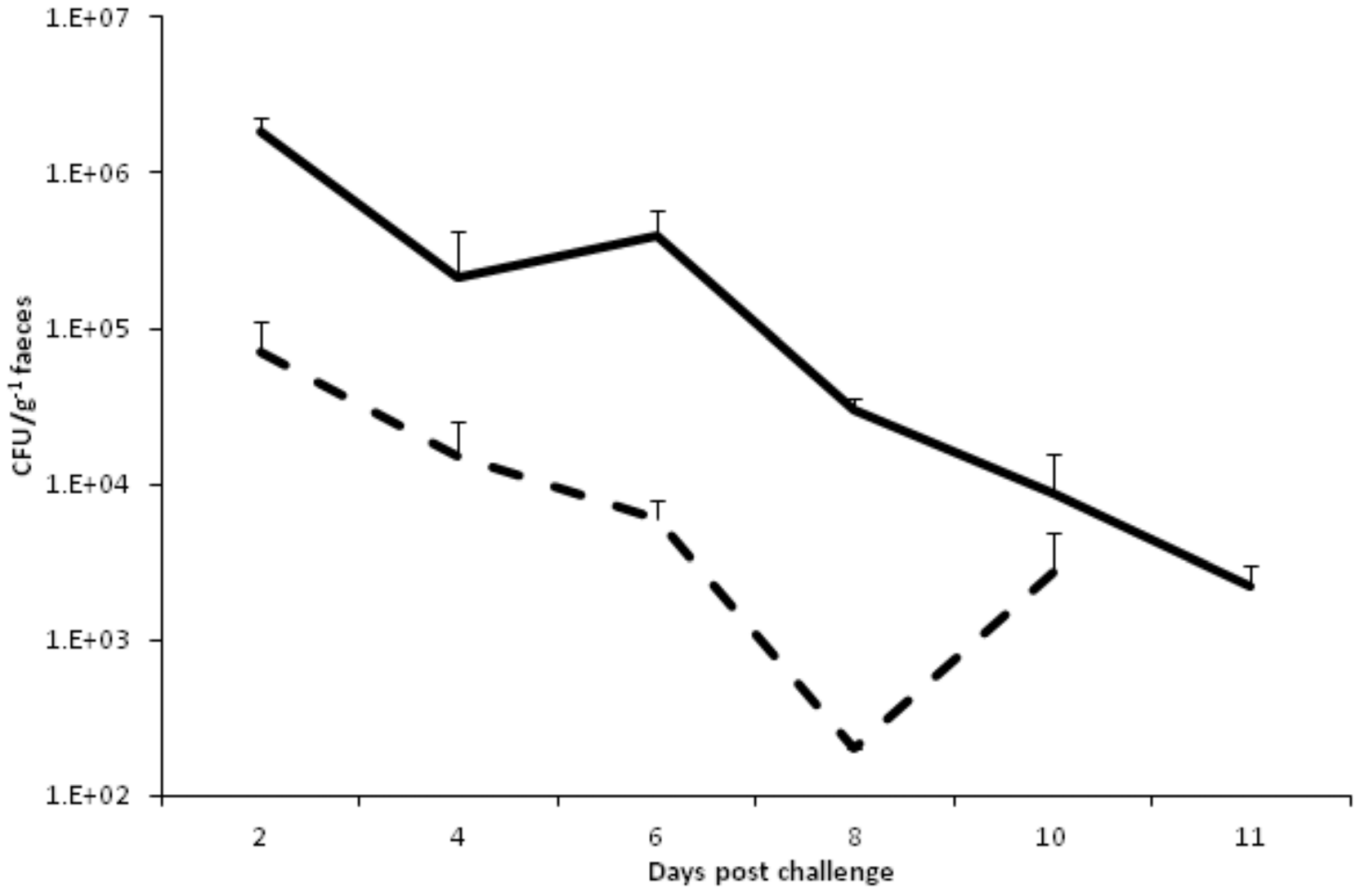
Feacal shedding of *C. difficile* spores. *C. difficile* recovery from faeces of surviving hamsters challenged with either CD1342 (full line) or M68 (dotted line). Bacterial faecal recoveries represent the geometric mean of 2 biological repeats, where each experiment used 3 animals per time point, plus the standard error of the mean (SEM).

Shedding profiles of either CD1342 or M68 were obtained from the faeces of animals that survived *C. difficile* challenge. High numbers of CD1342 were recovered from the faeces 2-d.p.c. (c. 1.8×10^6^ CFU/g^−1^ faeces) followed by a slow decrease with small numbers of spores recovered at 11-d.p.c. (c. 2.2×10^3^ CFU/g^−1^ faeces) (Figure 2). Animals challenged with M68 also peaked 2-d.p.c. although recoveries were at least 2 LOG lower initially (c. 7.2×10^4^ CFU/g^−1^ faeces) compared to CD1342 (Figure 2).

### Colonisation kinetics non-toxic of CD1342

**Figure 3:**
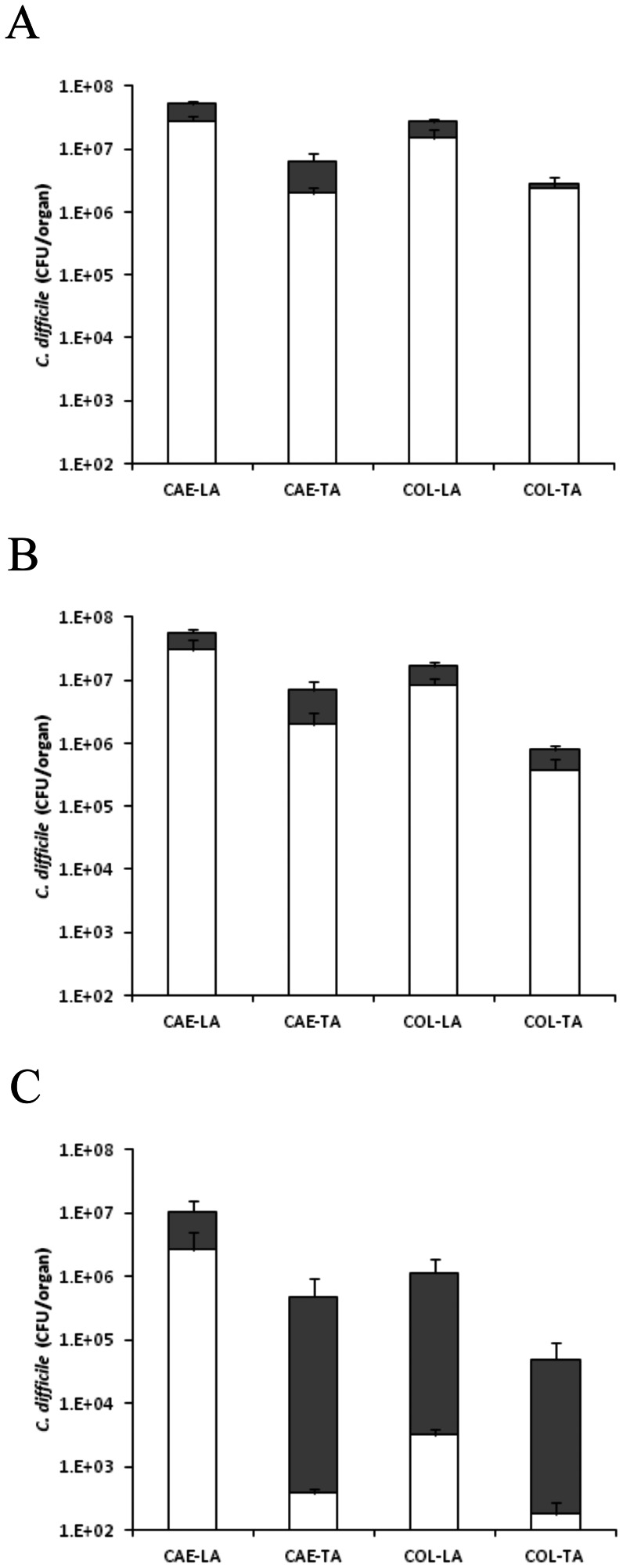
Colonisation kinetics of *C. difficile* CD1342 in hamsters. To monitor colonisation throughout the infection process hamsters were culled at 1- (A), 3- (B) and 11 (C) days post challenge. *C. difficile* was recovered from the caecum (CAE) and the colon (COL) either associated with the lumen (LA) or the tissue (TA). Filled bars represent vegetative bacteria whilst empty bars indicate bacteria in spore form. Bacterial recoveries represent the geometric mean plus the standard error of the mean (SEM) of 2 biological replicates, where a total of at least 5 animals were used per time interval.

To assess the colonisation kinetics of *C. difficile* CD1342 after inoculation, hamsters were culled at 1-, 3- & 11-d.p.c. to quantify bacterial levels in the caecum (CAE) and colon (COL). One-day after challenge with 10^4^ spores/animal, total *C. difficile* caecum levels reached approximately 5.7×10^7^ CFU/organ with higher bacterial levels in the lumen (-LA) compared to those bacteria more intimately associated with the tissue (-TA) (5.2×10^7^ & 5.0×10^6^ CFU/organ, CAE-LA & CAE-TA, respectively) (Figure 3A). Levels recovered from the colon were similar compared to the caecum (Figure 3A). At 1- & 3-d.p.c. the percentage of spores present was high representing 54 (CAE-LA), 39 (CAE-TA), 83 (COL-LA) & 80% (COL-TA) of total bacteria isolated, respectively in both tissues (Figure 3B), whilst at the experimental end-point (11-d.p.c.) the total number of bacteria isolated generally decreased by ∼1 LOG CFU/organ across all organ sites (Figure 3C).

### Colonisation kinetics of M68

**Figure 4:**
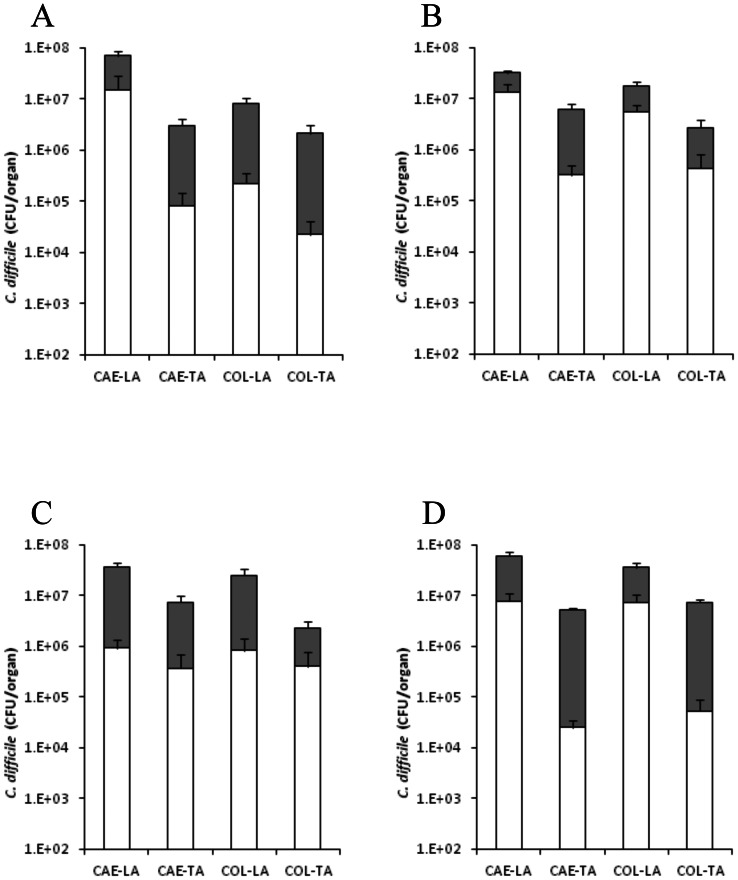
Colonisation kinetics of *C. difficile* M68 in hamsters. To monitor colonisation throughout the infection process hamsters were culled at 1- (A), 3- (B) and 11 (D) days post challenge and if animals succumbed to infection (C). *C. difficile* was recovered from the caecum (CAE) and the colon (COL) either associated with the lumen (LA) or the tissue (TA). Filled bars represent vegetative bacteria whilst empty bars indicate bacteria in spore form. Bacterial recoveries represent the geometric mean plus the standard error of the mean (SEM) of 2 biological replicates, where a total of at least 5 animals were used per time interval.

To measure the colonisation kinetics of *C. difficile* M68 in the caecum and colon, hamsters were culled at 1-, 3- & 11-d.p.c. and additionally if the animals succumbed to infection. At 1-d.p.c., caecum levels reached approximately 7.3×10^7^ CFU/organ whilst levels in the colon were slightly less, 8.8×10^6^ CFU/organ (Figure 4A). Again bacterial recoveries were higher in the lumen than those more intimately associated with the tissues. By 3-d.p.c. the pattern of colonisation remained similar to that seen on day 1, although the levels of spores recovered from most tissues increased (Figure 4B). The recoverable bacterial levels of those animals that succumbed to infection (45%; ∼83 h post challenge) were high across all tissues sites sampled, although showing no significant difference to those animals culled at a similar time point (3-d.p.c.), which showed no clinical symptoms (Figure 4C). At experimental end-point (11-d.p.c.), surviving hamsters had high *C. difficile* levels in all tissue sites however there was a c.a. ∼1 LOG reduction especially in the organisms more intimately associated with the tissues (Figure 4D).

### Colonisation kinetics of BI-7

**Figure 5:**
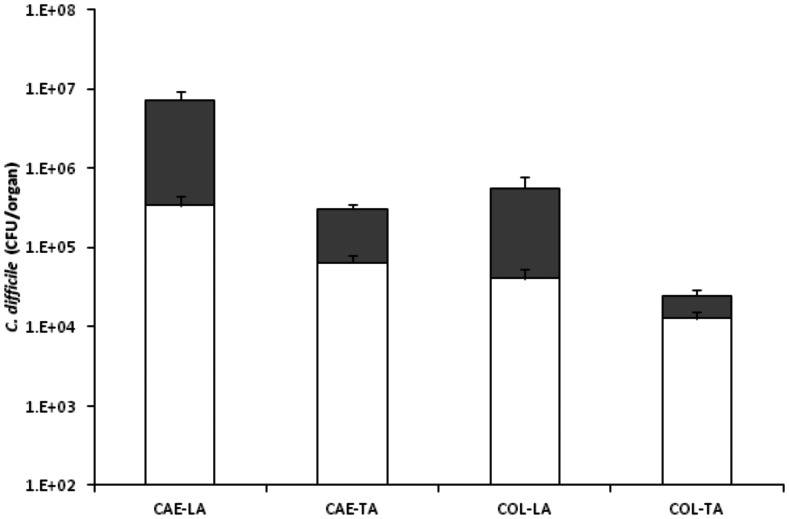
Colonisation kinetics of *C. difficile* BI-7 in hamsters. Bacteria recovered from hamsters challenged with BI-7 at time of cull (∼26 h). *C. difficile* was recovered from the caecum (CAE) and the colon (COL) either associated with the lumen (LA) or the tissue (TA). Filled bars represent vegetative bacteria whilst empty bars indicate bacteria in spore form. Bacterial recoveries represent the geometric mean plus the standard error of the mean (SEM), where a total of at least 5 animals were used.

Hamsters challenged with BI-7 resulted in a rapid fatal infection after ∼26 h. Total bacteria recovered at this time were significantly lower by at least 1 LOG than either CD1342 & M68 at the same time point (1-d.p.c.) with caecal recoveries highest compared to colon (6.9×10^6^, 3.1×10^5^, 5.6×10^5^ & 2.5×10^4^ CFU/organ; CAE-LA, CAE-TA, COL-LA & COL-TA, respectively; *p*≤0.0061 for all tissues vs both CD1342 & M68) (Figure 5). Levels of spores recovered were significantly lower compared to those seen with CD1342 at 1-d.p.c. (*p* = 0.0003 all tissues) (Figure 5).

### *In vivo* toxin levels

**Figure 6:**
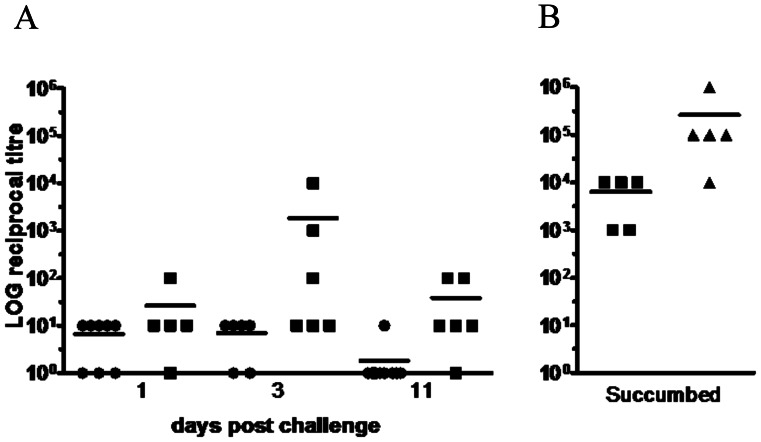
Cytotoxicity of caecal contents from hamsters challenged with *C. difficile* strains. *C. difficile* CD1342 (open circles), M68 (closed squares) or BI-7 (closed triangles) at either 1-, 3- & 11-d.p.c (A) or if the hamsters succumbed to infection (B). Toxin was quantified using a cell-based assay by serially diluting filtered caecal content obtained at *post-mortem*. Symbols represent mean toxin levels.

*In vivo* toxin activity was measured semi-quantitatively *in vitro* using a Vero cell toxicity assay by serially diluting filtered caecal luminal contents, and calculating the maximum fold-dilution at which cell toxicity was still detected (cell rounding). At each time point tested, little cell rounding activity was detected in the caecal filtrate from animals challenged with CD1342 (Figure 6A). Challenge with M68 resulted in a wide spread of caecal toxin measurements with peak toxin detected at 3-d.p.c. (Figure 6A). Hamsters that succumbed to infection with M68, ∼3-d.p.c., were associated with increased caecal toxin levels however these data were not significantly different compared to those hamsters which were culled at day 3 (*p* = 0.0568) (Figure 6B). The difficulty with this data is it is not possible to differentiate between those animals culled at 3-d.p.c. that may have subsequently succumbed to infection compared to those that would have survived. In comparison to animals that succumbed to challenge with M68, animals infected with BI-7 and culled at ∼26 h showed significantly higher levels of toxin activity (∼LOG 3.8 vs 5.4; *p* = 0.0215) (Figure 6B).

### Histological changes

**Figure 7:**
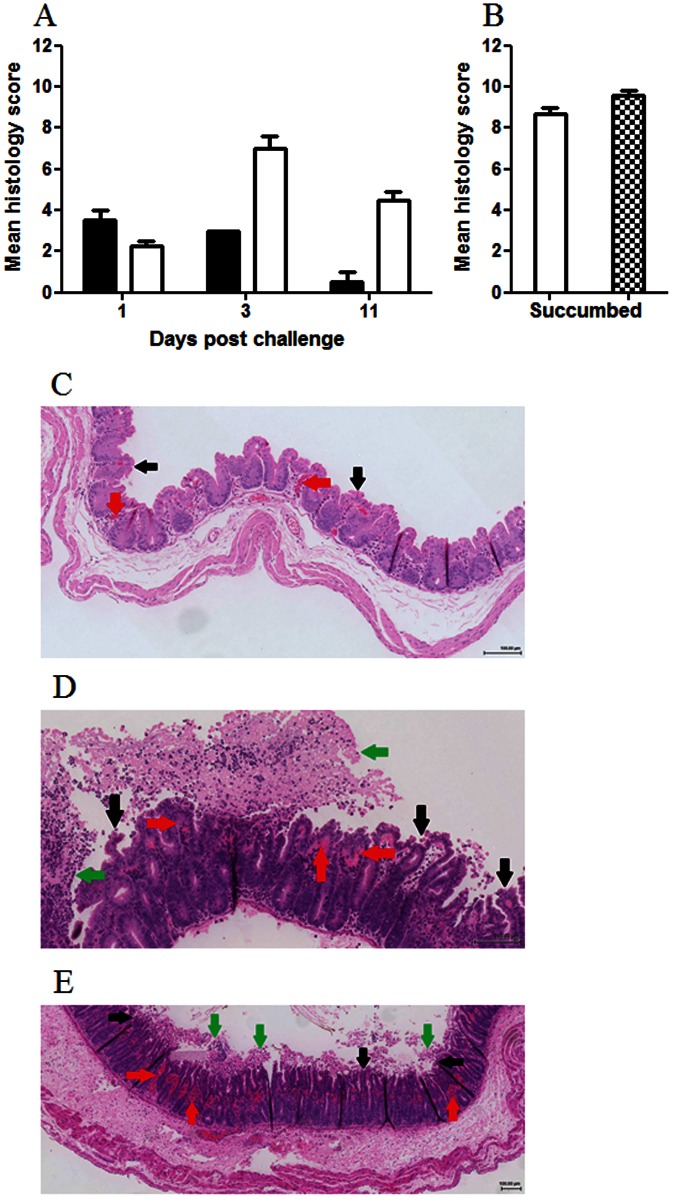
Mean histology scores from hamsters challenged with *C. difficile* strains. *C. difficile* CD1342 (filled columns), M68 (open columns) or BI-7 (checked column) at either 1-, 3- & 11-d.p.c (A) or if the hamsters succumbed to infection (B). Caecal pathology was graded by neutrophil margination, haemorrhagic congestion, hyperplasia and percent barrier involvement from at least four animals. Typical caecal histology from hamsters challenged with either CD1342 (at 1-d.p.c. - C), M68 (at 3-d.p.c. - D) or BI-7 (at ∼26 h - E). Red arrows denotes unclotted red blood cells within the villus structure; black arrows denotes epithelial barrier destruction & green arrows denotes transmural neutrophil infiltrate.

Considering the lack of clinical symptoms, histological analysis of the caeca from hamsters challenged with CD1342 and sacrificed 1-d.p.c. showed surprising changes; whilst the epithelial layer seemed intact unclotted red blood cells were associated within the villus structure (Figure 7A & C). Accompanying this haemorrhagic congestion was an increase in circulating submucosal neutrophil cells. Similar caecal pathology was also observed in all hamsters infected with CD1342 and culled at 3-d.p.c. By 11-d.p.c. hamsters challenged with CD1342 showed no caecal pathology, tissue was similar to uninfected hamsters (Figure 7). Similarly, animals challenged with M68 and culled 24 h later showed a modest increase in circulating neutrophils and increased numbers of red blood cells within the capillaries. Whilst animals culled at 3-d.p.c. showed typical characteristic pathology exemplified by high epithelial cell loss, transmural neutrophil infiltrate and high levels of unclotted red blood cells associated with the villus structure and the lumen (Figure 7A & D). Intestinal pathology was most pronounced at 3-d.p.c. but intestinal epithelial cell loss and inflammatory cell infiltration persisted until experimental end-point (11-d.p.c.) (Figure 7). In addition, these animals showed tissue hyperplasia in the terminal colon that persisted to day 11 (data not shown). Hamsters that succumbed to infection with M68, showed more caecal pathology in comparison to those lacking symptoms and culled at 3-d.p.c. Typically more unclotted red blood cells was associated with the tissue and severe epithelial cell loss was apparent (Figure 7B). At clinical end-point, caecal tissue from hamsters challenged with BI-7 showed pathological changes typically associated with *C. difficile* infection (Figure 7B & E). Caecal tissue showed high levels of transmural neutrophil infiltrate and unclotted red blood cells with wide-ranging epithelial cell loss and extensive damage to the villus structure.

## Discussion

Here we present a detailed virulence study of three human isolates of *C. difficile*, BI-7, M68 and CD1342, in the hamster model of infection. Antimicrobial susceptibility assays showed both M68 and CD1342 had high-level resistance to clindamycin (>256 µg/ml), whilst BI-7 had an intermediate level of resistance (64 µg/ml). Clindamycin is an important clinical antibiotic that has been implicated with induction of CDI [29] with many strains showing high resistance including all A−B+ strains isolated from South Korean CDI cases [30]. Previously, we described the infection kinetics of a clindamycin-sensitive Ribotype 027 U.K. isolate, R20291 (clindamycin MIC = 8 µg/ml), where 100% of hamsters succumbed to infection with a mean time of 46.7 h [24]. The much faster infection kinetics displayed by the closely related Ribotype 027, BI-7, probably reflects the more efficient germination and subsequent survival of this strain within the clindamycin treated environment of the hamster gut. Due to this high-level clindamycin resistance hamsters were challenged with *C. difficile* one-day prior to clindamycin infection without any detrimental effects on inocula.

Challenge of hamsters with *C. difficile* strain CD1342 resulted in a 100% colonisation rate, with animals remaining colonised until the end of the study 14-d.p.c. This is similar to other studies with non-toxigenic strains where colonisation was observed until at least 31-d.p.c. [31]. Although PCR detection for the presence of the PaLoc and for the CDT-encoding genes in CD1342 were negative (data not shown), this strain still caused mild caecal pathology characterised by local acute epithelial cell loss, haemorrhagic congestion and neutrophil cell influx. This suggests that, at least in this strain, *C. difficile* CD1342 possesses an as yet uncharacterised virulence factor that is able to cause cell loss and damage, activating the immune system in the process. In the absence of the dominating effects of the toxins, i.e. using naturally occurring non-toxic strains, the hamster model of infection is ideally suited to elucidating potential transmission and/or colonisation factors for *C. difficile* infection. Sequencing of these types of non-toxic strains may give us more insight to such factors [32].

Bacterial germination and outgrowth of CD1342 was rapid within the animal as even assuming 100% germination from the 10^4^ spores used to challenge the animals bacteria in the caecum and colon had multiplied by at least ∼3 LOG CFU within the first 24 h. High levels of spores were also observed, demonstrating the rapid *in vivo* germination, replication, sporulation and shedding of this strain in this short timeperiod. The rapid growth and high rates of shedding, thus enhanced transmission potential, coupled with potential acquisition of virulence factors could result in new clades of *C. difficile* with enhanced virulence similar to that observed with ribotype 027 strains, resulting in new global epidemics. The intestinal environment is a ‘hotbed’ for genetic exchange mediated by bacteriophages [33] and these exchanges have resulted in the acquisition of virulence factors like antibiotic resistance determinants and pathogenicity islands in several bacterial species, such as the LEE locus in enterohemorrhagic *E. coli* [34]. This type of genetic transfer has potential implications for the introduction of toxin encoding determinants, such as the PaLoc, to be transferred to previously non-toxic strains. This has potential implications on the potential use of non-toxic strains as probiotics to toxic *C. difficile* [31], [35]

When challenged with the toxin A negative *C. difficile* strain, M68, 100% of hamsters were colonised and shed this strain, with 45% of hamsters succumbing to disease. As all hamsters showed classic symptoms of CDI (wet tail) before potential recovery, the use of the telemetry system proved invaluable as we were able to discriminate those animals that had transitory disease from those that rapidly succumbed. These data show that, similar to a clinical setting, strain M68 (only producing a functional TcdB) is not only able to cause disease but can cause lethality in an *in vivo* model, which suggests that TcdA is not essential for disease initiation. However, with only a 55% survival rate in hamsters challenged with this strain, there may be a role for TcdA in fulminant CDI with this strain. Whilst it is not possible to directly attribute the role of individual genes in this type of study, the use of isogenic mutants to clarify the role of specific toxins has also been subject to controversy. In particular there has been confusion over the role of TcdA in CDI with isogenic mutants of *C. difficile* strain 630 where toxin genes have individually been disrupted generating conflicting data. Lyras *et al.* [36] first reported a minimal role for TcdA whilst Kuehne *et al.* [37] found an essential role for TcdA in CDI in the hamster. These differences may reflect the technologies used in mutant generation, differences in SNP profiles between the strains from different labs and even the method used to determine endpoint of experiments using the animal model. In contrast, our data is similar to that reported by [38], where hamsters colonised with an A−B+ clinical isolate (CF2) had a 50% survival rate. This suggests the role for TcdA in pathogenesis may vary dependent on strain and experimental conditions. Differences may reflect the acceptable endpoints criteria in different countries. However, within this experiment in which the endpoint is more refined a mixed picture is observed. Why some animals succumb to infection with M68 is unclear at present. Those animals that succumbed to infection showed no significant differences in total bacterial organ recoveries compared to animals culled at a similar time (3-d.p.c.). The small increase in vegetative cells observed could produce more TcdB, as shown by the toxin assay, causing more epithelial damage compared to those animals that survive. Interestingly those animals that survived challenge with M68, typically displayed an elevated core temperature above the normal range. Macrophages exposed to either TcdA or TcdB have been shown to release interleukin 1β (IL-1β) [39], a key cytokine that, along with IL-6, can cause an increase in body temperature in rodents [40]. Patients with severe *C. difficile* colitis often display clinically elevated IL-1β in their stool samples [41]. Such an increased cytokine profile could be responsible for the febrile response seen in surviving animals. This possibility questions whether the surviving hamsters were better able to mount an appropriate immune response to toxin exposure, maybe due to genetic differences as the hamsters used in this study are from an out-bred colony.

Since the initial Canadian outbreak [42], Ribotype 027 isolates have spread globally, causing major outbreaks in almost every continent. These strains have been associated with increased disease severity & reoccurrence rates, leading to this group of strains to be classed as ‘hypervirulent’ [43], [9]. When challenged with BI-7 100% of hamsters rapidly succumbed to disease, ∼26 h post challenge. In a similar study, Razaq *et al.* [44] observed increased mortality rates with epidemic Ribotype 027 isolates, especially with a clindamycin resistant isolate. Histological analysis of hamsters challenged with BI-7 showed severe epithelial cell loss, transmural neutrophil infiltrate and extensive damage to the submucosal structure. Such damage is, at least in part, due to the high toxin titres seen from the tissue samples, which considering the modest level of recovered bacteria is surprising. This high toxin titre could be due to more toxin being produced or the produced toxin is a more efficient enzyme due to changes in the protein sequence [45]. Recently Lanis *et al.* [46] showed that TcdB from strain R20291 (Ribotype 027) was more toxic due to conformational changes that occurred at a higher pH when compared to TcdB from strain 630. Another explanation for this rapid mortality could be the presence of the binary toxin, CDT. Through the induction of microtubule-based protrusions, CDT may enhance the adherence of *C. difficile* to host cells [47].

In conclusion, using the hamster model of infection we characterised the infectivity profiles of three *C. difficile* isolates varying in toxin carriage. The toxin negative strain colonised hamsters and was shed with high efficiency but caused tissue damage, however no clinical symptoms were observed during the infection process. Whereas the toxin A negative strain caused mortality in 55% of hamsters, potentially associated with the increased toxin detected. Those animals that survived challenge displayed a febrile response highlighting potential host genetic differences involved in survival of CDI. Challenge with *C. difficile* BI-7 resulted in a rapid fatal infection in 100% of the animals, causing extensive tissue damage and high toxin titres observed.
